# PMBC: a manually curated database for prognostic markers of breast cancer

**DOI:** 10.1093/database/baae033

**Published:** 2024-05-15

**Authors:** Jiabei Liu, Yiyi Yu, Mingyue Li, Yixuan Wu, Weijun Chen, Guanru Liu, Lingxian Liu, Jiechun Lin, Chujun Peng, Weijun Sun, Xiaoli Wu, Xin Chen

**Affiliations:** School of Automation, Guangdong University of Technology, 100 Outer Ring West Road, Guangzhou University City, Panyu District, Guangzhou 510006, China; School of Automation, Guangdong University of Technology, 100 Outer Ring West Road, Guangzhou University City, Panyu District, Guangzhou 510006, China; School of Automation, Guangdong University of Technology, 100 Outer Ring West Road, Guangzhou University City, Panyu District, Guangzhou 510006, China; School of Automation, Guangdong University of Technology, 100 Outer Ring West Road, Guangzhou University City, Panyu District, Guangzhou 510006, China; School of Automation, Guangdong University of Technology, 100 Outer Ring West Road, Guangzhou University City, Panyu District, Guangzhou 510006, China; School of Automation, Guangdong University of Technology, 100 Outer Ring West Road, Guangzhou University City, Panyu District, Guangzhou 510006, China; School of Automation, Guangdong University of Technology, 100 Outer Ring West Road, Guangzhou University City, Panyu District, Guangzhou 510006, China; School of Automation, Guangdong University of Technology, 100 Outer Ring West Road, Guangzhou University City, Panyu District, Guangzhou 510006, China; School of Automation, Guangdong University of Technology, 100 Outer Ring West Road, Guangzhou University City, Panyu District, Guangzhou 510006, China; School of Automation, Guangdong University of Technology, 100 Outer Ring West Road, Guangzhou University City, Panyu District, Guangzhou 510006, China; Guangdong Key Laboratory of IoT Information Technology, Guangdong University of Technology, 100 Outer Ring West Road, Guangzhou University City, Panyu District, Guangzhou 510006, China; School of Biomedical and Pharmaceutical Sciences, Guangdong University of Technology, 100 Outer Ring West Road, Guangzhou University City, Panyu District, Guangzhou 510006, China; School of Automation, Guangdong University of Technology, 100 Outer Ring West Road, Guangzhou University City, Panyu District, Guangzhou 510006, China

## Abstract

Breast cancer is notorious for its high mortality and heterogeneity, resulting in different therapeutic responses. Classical biomarkers have been identified and successfully commercially applied to predict the outcome of breast cancer patients. Accumulating biomarkers, including non-coding RNAs, have been reported as prognostic markers for breast cancer with the development of sequencing techniques. However, there are currently no databases dedicated to the curation and characterization of prognostic markers for breast cancer. Therefore, we constructed a curated database for prognostic markers of breast cancer (PMBC). PMBC consists of 1070 markers covering mRNAs, lncRNAs, miRNAs and circRNAs. These markers are enriched in various cancer- and epithelial-related functions including mitogen-activated protein kinases signaling. We mapped the prognostic markers into the ceRNA network from starBase. The lncRNA NEAT1 competes with 11 RNAs, including lncRNAs and mRNAs. The majority of the ceRNAs in ABAT belong to pseudogenes. The topology analysis of the ceRNA network reveals that known prognostic RNAs have higher closeness than random. Among all the biomarkers, prognostic lncRNAs have a higher degree, while prognostic mRNAs have significantly higher closeness than random RNAs. These results indicate that the lncRNAs play important roles in maintaining the interactions between lncRNAs and their ceRNAs, which might be used as a characteristic to prioritize prognostic lncRNAs based on the ceRNA network. PMBC renders a user-friendly interface and provides detailed information about individual prognostic markers, which will facilitate the precision treatment of breast cancer. PMBC is available at the following URL: http://www.pmbreastcancer.com/.

## Introduction

Breast cancer is the most commonly diagnosed cancer and has the highest mortality in women, according to Global Cancer Statistics 2020 (1). As a highly heterogeneous disease, subtype-specific therapeutic strategies were utilized to improve the survival of patients. However, de novo resistance to therapies or acquired resistance always led to unfavorable outcomes.

Numerous biomarkers have been identified and validated to predict the survival of patients since the wide application of microarrays and the next generation of sequencing. A 70-gene signature has been proposed for prognosis prediction and widely applied to select therapeutic strategies (2). Oncotype DX is a prognostic signature for hormone-receptor–positive breast cancer based on 21 genes (3). EndoPredict combines the expression of proliferative and etrogen reptor signaling–associated genes for the distant recurrence of breast cancer (4). The PAM50 signature was developed to identify the intrinsic subtype and recurrence risk of breast cancer (5).

Moreover, various tools have been developed to predict the prognostic potential of genes or non-coding RNAs in various cancers, including breast cancer, such as Survival Genie (6), miRpower (7), PrognoScan (8), G-DOC (9, 10), GOBO (11), SurvExpress (12), BreastMark (13), SurvMicro (14), Kaplan–Meier Plotter (15), the cBio Cancer Genomics Portal (http://cbioportal.org) (16), PROGgeneV2 (17), KM-Express (18) and GEPIA2 (19). These tools have greatly facilitated the identification and validation of prognostic markers. However, there is currently no database for curated prognostic markers of breast cancer, including both protein-coding and non-coding RNAs. Therefore, we constructed the PMBC, a manually curated database for prognostic markers of breast cancer. PMBC contains 1070 prognostic biomarkers from 714 articles in PubMed. Users can browse and search prognostic markers of their interests. All biomarkers are easy to download from http://www.pmbreastcancer.com/.

## Materials and methods

### Data acquisition

We searched in PubMed (http://www.ncbi.nlm.nih.gov/pubmed) for publications with prognostic markers related to breast cancer. Specifically, we used the following search terms: ‘((prognostic [Title/Abstract] OR prognosis [Title/Abstract]) AND breast [Title/Abstract]) AND (cancer [Title/Abstract] OR tumor [Title/Abstract] OR carcinoma [Title/Abstract]) AND (“2010/01/01”[Date - Publication]: “2022/04/01”[Date - Publication])’. We only focus on the publications in the journal with impact factors >5.0 for further curation.

### CeRNA network

We downloaded the ceRNAs for mRNAs, lncRNAs and pseudogenes from starBase v2.0 (http://starbase.sysu.edu.cn/) using the Web API with default parameters. In total, the ceRNA network was obtained, comprising 8266 ceRNA pairs and 18 942 RNAs.

### Network topological analysis

The R package igraph was used to calculate the topological features of RNAs in the ceRNA network (R 4.1.2). The degree of an RNA is the number of its neighboring RNAs in the ceRNA network. The closeness centrality of one RNA *i* is calculated as follows: ${C_i} = \frac{{n - 1}}{{\sum\nolimits_{i \ne j} {{d_{ij}}} }}$, where nodes *j* is a different node from node *i*, ${d_{ij}}$ denotes the distance between node *i* and *j, n* is the total number of nodes in the ceRNA network. The betweenness of one RNA *i* is calculated as following: ${B_i} = \mathop \sum \limits_{s \ne i \ne t} \frac{{{\delta _{st}}\left( i \right)}}{{{d_{st}}}}$, where RNAs *s* and *t* are nodes different from RNA *i* in the ceRNA network, ${d_{st}}$ denotes the shortest paths from RNA *s* to *t*, ${\delta _{st}}$ denotes the number of shortest paths from RNA *s* to *t* which RNA *i* lies on. For two RNAs *s* and *t*, the ratio is the percentage of shortest path that RNA *i* lies on. The sum of all RNA pairs is the betweenness of RNA *i*. For RNA permutations, equal numbers of prognostic RNAs were randomly selected from all the RNAs in the human genome with replacement for 100 000 times. Cytoscape v 3.8.0 was used to visualize the ceRNA network.

### Database construction

The database is freely available at http://www.pmbreastcancer.com/. The database was developed using Tomcat (v 9.0.0) as the Web server and using Asynchronous Javascript And XML (AJAX) technology to realize the front-end and back-end interactions. The MySQL (v 5.5) data server was used to store and manage the datasets. The creation of result tables was performed using jQuery (v3.6.0) plugin software. The website is supported on popular web browsers, such as Microsoft Edge, Google Chrome and Firefox.

### Technical details

We used HyperText Markup Language for the webpage structure, CSS for graphic design and JavaScript with AJAX for interactive elements. We utilized AJAX technology for the fore-end to execute front-end development solutions, handling asynchronous requests through the $.ajax() method in the jQuery framework. For the back-end, we used Servlet to implement the back-end development solutions, selecting Tomcat as the web server to route the front-end requests to the corresponding Servlet based on the URL paths defined in the $.ajax() method. We connected the web-based platform with the database of breast cancer prognostic markers with MySQL for querying or inserting data and generating JavaScript Object Notation data in response to return it to system users.

## Results

### Data source

We manually curated 714 publications and obtained 1070 prognostic markers appearing in 1357 records for breast cancer. As shown in Table 1, 81.21% (869/1070) of the prognostic markers belongs to mRNA, 6.54% (70/1070) to lncRNA, 9.06% (97/1070) to miRNA, 2.99% (32/1070) to circRNA and 0.19% (2/1070) to pseudogene.

**Table 1.** Number of RNA records and unique RNA markers

	Number of records	Number of unique markers
mRNA	1089	869
lncRNA	80	70
miRNA	136	97
circRNA	49	32
Pseudogene	3	2

### Basic statistics of the curated prognostic markers

**Figure 1.**
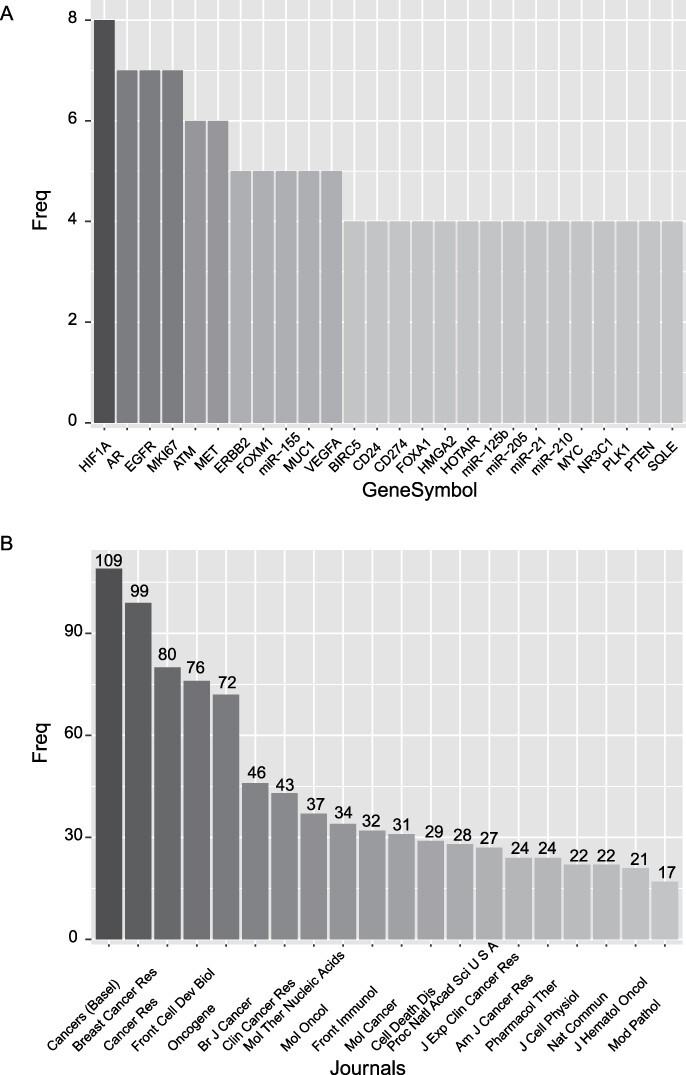
Basic statistics of the curated prognostic markers. (A) Most frequently reported prognostic markers. (B) The journals with the top number of prognostic markers.

Among the 1070 prognostic markers of breast cancer, we sorted them according to their frequency. As shown in Figure 1A, HIF1A is the most frequently reported prognostic marker, and AR, EGFR and MIK67 are the second most reported markers. HOX transcript antisense RNA is the most frequently reported prognostic lncRNA, while miR-125b, miR-205, miR-21 and miR-210 are the most frequently reported prognostic miRNAs. Besides, we rank the journals according to the number of reported prognostic markers (Figure 1B). The journal ‘Cancers (Basal)’ is at the top of the list, followed by the journals ‘Breast Cancer Res’ and ‘Cancer Res’.

### Database design

**Figure 2.**
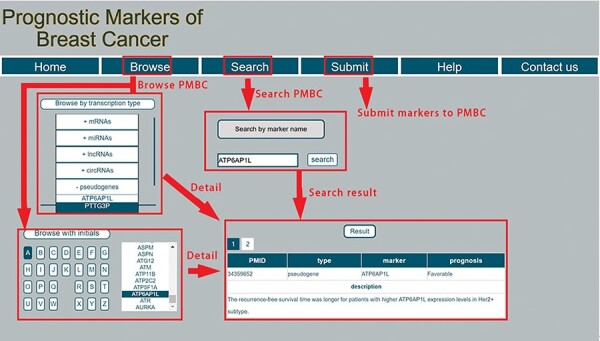
A schematic workflow of PMBC.

PMBC provides a user-friendly interface to allow users to easily query information for prognostic markers of breast cancer. A screenshot of the database for prognostic markers of breast cancer is shown in Figure 2. Specifically, the webpage consists of seven parts: ‘Home’, ‘Browse’, ‘Search’ ‘Submit’, ‘Help’ and ‘Contact us’. In the ‘Browse’ page, users can browse prognostic markers by RNA type, including miRNA, lncRNA, mRNA, circRNA and pseudogene. PMBC also provides browsing with initials of prognostic markers. By clicking a specific initial, all prognostic markers with this initial are returned and shown in a table. In the ‘Search’ page, PMBC allows users to search by symbols of prognostic markers. The database enables fuzzy searching, allowing users to return the most possible matching results. The corresponding results will be returned about the searched prognostic marker, including the publication ID in the PubMed database of National Center for Biotechnology Information, the RNA type of the marker, the favorable or poor outcome for patients with high expression of the marker and the detailed description in the literature. PMBC designed a ‘Submit’ page that enables researchers to submit up-to-date prognostic markers of breast cancer. Once approved by the review committee, the background database will be updated including the submitted record. Moreover, a step-by-step tutorial is also rendered to facilitate users quickly know how to use the database in the ‘Help’ page. The users can also contact us with the information provided in the ‘Contact us’ page.

### Function enrichment

**Figure 3.**
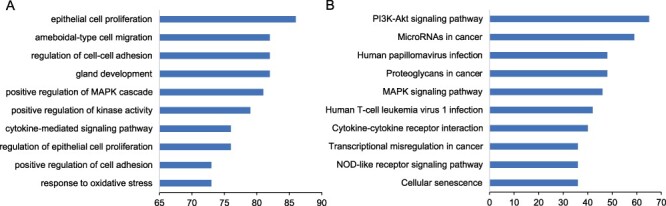
Functional enrichment of prognostic markers. We performed the functional enrichment of the known prognostic RNAs in biological processes of Gene Ontology (GO) and Kyoto Encyclopedia of Genes and Genomes (KEGG) pathway. The significant terms were ranked according to the number of prognostic markers. The top 10 significant terms were shown for (A) GO and (B) KEGG.

We performed functional enrichment to explore the biological processes and pathways for the known prognostic markers. The results showed that these markers were enriched in ‘regulation of epithelial cell proliferation’, ‘positive regulation of mitogen-activated protein kinases (MAPK) cascade’, ‘gland development’, and ‘epithelial cell proliferation’ (Figure 3A). Among the significant pathways, the pathways enriched by the greatest number of prognostic markers are ‘Proteoglycans in cancer’, ‘MicroRNAs in cancer’, ‘PI3K-Akt signaling pathway’ and ‘MAPK signaling pathway’ (Figure 3B).

### Known prognostic markers play pivotal roles in the ceRNA network

The mRNAs and lncRNAs compete to bind with miRNAs and function as ceRNAs. Therefore, we systematically characterize prognostic markers of breast cancer based on the ceRNA network. These prognostic markers were mapped to the downloaded ceRNA network, which comprises mRNA, lncRNA and pseudogene. As a result, 631 markers have interactions in the ceRNA network, including 602 mRNAs, 28 lncRNAs and one pseudogene.

**Figure 4.**
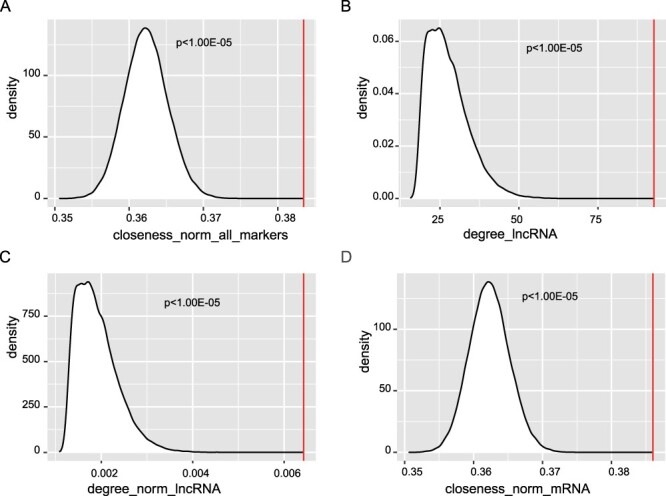
Significant topological features of prognostic markers. We compared the topological value of the known prognostic RNAs with that of the random RNAs. (**A**) The normalized closeness was compared with that of random. The raw (B) and normalized (**C**) degree of prognostic lncRNAs was compared with that of random. (**D**) The normalized closeness of prognostic mRNAs was compared with that of random.

Then, we characterize the topological features of these prognostic markers from the perspectives of degree, closeness and betweenness. The RNAs (nodes) in the ceRNA network were ranked according to these three topological features, respectively. By comparing the topological value of the prognostic RNAs with that of the random RNAs, we found that known prognostic RNAs have higher normalized closeness than random (*P* < 1.00E − 05, Table 2, Figure 4A), which suggests that they have a shorter distance to other nodes in the ceRNA network. Besides, we divided the prognostic markers into three RNA types, lncRNA, mRNA and pseudogene. Compared with the random RNAs, prognostic lncRNAs have both higher raw and normalized degrees, (*P* < 1.00E − 05, Table 2, Figure 4B and C). For prognostic mRNAs, they have significantly higher normalized closeness (*P* < 1.00E − 05, Table 2, Figure 4D). From these results, we conclude that the lncRNAs play important roles in maintaining the interactions between lncRNAs and their ceRNAs, which might be used as a characteristic to prioritize prognostic lncRNAs based on its ceRNA network.

**Table 2.** Topological features of prognostic markers

	Random	All markers	lncRNA	mRNA	Pseudogene
degree_raw	3.29E + 01	3.05E** + **01 (*P* = 0.28)	**9.27E + 01** (*P* < 1.00E-5)	2.76E + 01 (*P* = 0.43)	3.00 (*P* = 1.00)
degree_norm	2.28E − 03	2.10E − 03 (*P* = 0.28)	**6.42E** − **03** (*P* ≤ 1.00E-5)	1.91E − 03 (*P* = 0.43)	2.08E − 04 (*P* = 1.00)
betweenness_raw	2.20E + 04	1.26E + 04 (*P* = 0.39)	2.32E + 04 (*P* = 0.14)	1.22E + 04 (*P* = 0.41)	7.95E − 01 (*P* = 1.00)
betweenness_norm	2.12E − 04	1.21E − 04 (*P* = 0.39)	2.23E − 04 (*P* = 0.14)	1.17E − 04 (*P* = 0.41)	0.00 (*P* = 1.00)
closeness_raw	3.20E − 03	2.70E − 05 (*P* = 0.97)	2.30E − 5 (*P* = 1.00)	2.70E − 05 (*P* = 0.97)	2.10E − 05 (*P* = 1.00)
closeness_norm	3.65E − 01	**3.83E** − **01** (*P* < 1.00E − 05)	3.3E − 01 (*P* = 1.00)	**3.86E** − **01** (*P* < 1.00E − 05)	3.00E − 01 (*P* = 1.00)

The topological features with *P* < 0.05 are marked in Bold.

**Figure 5.**
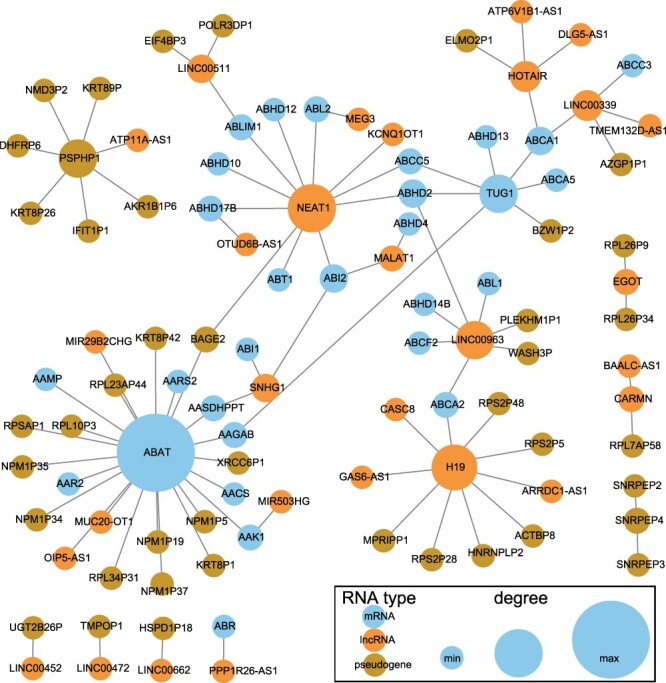
CeRNA network of prognostic markers. The blue nodes represent mRNAs, the orange nodes represent lncRNAs and the dark gold nodes represent pseudogenes. The node size is proportional to its degree in the ceRNA network.

We further extract the ceRNA subnetwork for the 631 prognostic markers, and each pair of markers competitively binds to same miRNAs. The network is shown in Figure 5. The lncRNA NEAT1 competes with 11 RNAs, including lncRNAs and mRNAs, to bind with miRNAs. Majority of the ceRNAs of ABAT belong to pseudogenes, while nearly all the ceRNAs of the pseudogene PSPHP1 are pseudogenes.

### Future extensions

We will review and select newly reported prognostic indicators for breast cancer every 6 months and update the database with user-submitted material monthly. In the current ceRNA network, there are no interactions for circRNA, which fail to characterize them in the ceRNA network. It will be our future direction to reconstruct the ceRNA network to include more RNA types, including but not limited to circRNA. The extended number of prognostic markers and incorporation of more comprehensive ceRNA interactions will improve the utility and coverage of this database. We will have a global and precise understanding of prognostic markers of breast cancer.

## Discussion

We have developed a user-friendly database, PMBC. PMBC provides curated prognostic markers for breast cancer. It is different from survival analysis web tools in that it (i) is composed of prognostic markers that are manually curated from publications, (ii) contains various types of RNA markers, including mRNA, miRNA, lncRNA, circRNA and pseudogene, which may not simultaneously be detected in one dataset, (iii) enables the characterization of prognostic markers from the perspective of the ceRNA network and (iv) facilitates the prioritization of prognostic markers based on the network topology of markers in PMBC. To ensure quick and easy searching ability, an intuitive querying interface was implemented in PMBC.

## Conclusions

PMBC hosts comprehensive prognostic markers spanning from mRNAs, lncRNAs, miRNAs, to circRNAs. Besides, it provides convenient and user-friendly interfaces to explore the known prognostic markers. We have generated PMBC as a cross-validation tool for researchers to identify potential prognostic RNA biomarkers to follow up for further research. PMBC allows for rapid retrieval and organization of information about cancer prognostic markers, facilitating researchers in comprehending pertinent data and enhancing therapy efficacy and patient well-being. PMBC will help accelerate translational research from bench to bedside.

## Data Availability

The data presented in this study are available in the article.

## References

[1] Sung H. , Ferlay J., Siegel R.L. et al. (2021) Global Cancer Statistics 2020: GLOBOCAN estimates of incidence and mortality worldwide for 36 cancers in 185 countries. *CA Cancer J. Clin*., 71, 209–249. 33538338 10.3322/caac.21660

[2] van ‘t Veer L.J. , Dai H., van de Vijver M.J. et al. (2002) Gene expression profiling predicts clinical outcome of breast cancer. *Nature*, 415, 530–536. 11823860 10.1038/415530a

[3] Paik S. , Shak S., Tang G. et al. (2004) A multigene assay to predict recurrence of tamoxifen-treated, node-negative breast cancer. *N. Engl. J. Med*., 351, 2817–2826. 15591335 10.1056/NEJMoa041588

[4] Filipits M. , Rudas M., Jakesz R. et al. (2011) A new molecular predictor of distant recurrence in ER-positive, HER2-negative breast cancer adds independent information to conventional clinical risk factors. *Clin. Cancer. Res*., 17, 6012–6020. 21807638 10.1158/1078-0432.CCR-11-0926

[5] Parker J.S. , Mullins M., Cheang M.C.U. et al. (2009) Supervised risk predictor of breast cancer based on intrinsic subtypes. *J. Clin. Oncol*., 27, 1160–1167. 19204204 10.1200/JCO.2008.18.1370 PMC2667820

[6] Dwivedi B. , Mumme H., Satpathy S. et al. (2022) Survival Genie, a web platform for survival analysis across pediatric and adult cancers. *Sci. Rep*., 12, 3069. 10.1038/s41598-022-06841-0 PMC8866543 35197510

[7] Lanczky A. , Nagy Á., Bottai G. et al. (2016) miRpower: a web-tool to validate survival-associated miRNAs utilizing expression data from 2178 breast cancer patients. *Breast Cancer Res. Treat*., 160, 439–446. 27744485 10.1007/s10549-016-4013-7

[8] Mizuno H. , Kitada K., Nakai K. et al. (2009) PrognoScan: a new database for meta-analysis of the prognostic value of genes. *BMC Med. Genet*., 2, 18. 10.1186/1755-8794-2-18 PMC2689870 19393097

[9] Madhavan S. , Gusev Y., Harris M. et al. (2011) G-DOC: a systems medicine platform for personalized oncology. *Neoplasia*, 13, 771–783. 21969811 10.1593/neo.11806 PMC3182270

[10] Bhuvaneshwar K. , Belouali A., Singh V. et al. (2016) G-DOC Plus—an integrative bioinformatics platform for precision medicine. *BMC Bioinf*., 17, 193. 10.1186/s12859-016-1010-0 PMC4851789 27130330

[11] Ringner M. , Fredlund E., Häkkinen J. et al. (2011) GOBO: gene expression-based outcome for breast cancer online. *PLoS One*, 6, e17911. 10.1371/journal.pone.0017911 PMC3061871 21445301

[12] Aguirre-Gamboa R. , Gomez-Rueda H., Martínez-Ledesma E. et al. (2013) SurvExpress: an online biomarker validation tool and database for cancer gene expression data using survival analysis. *PLoS One*, 8, e74250. 10.1371/journal.pone.0074250 PMC3774754 24066126

[13] Madden S.F. , Clarke C., Gaule P. et al. (2013) BreastMark: an integrated approach to mining publicly available transcriptomic datasets relating to breast cancer outcome. *Breast Cancer Res*., 15, R52. 10.1186/bcr3444 PMC3978487 23820017

[14] Aguirre-Gamboa R. and Trevino V. (2014) SurvMicro: assessment of miRNA-based prognostic signatures for cancer clinical outcomes by multivariate survival analysis. *Bioinformatics*, 30, 1630–1632. 24519378 10.1093/bioinformatics/btu087

[15] Gyorffy B. (2021) Survival analysis across the entire transcriptome identifies biomarkers with the highest prognostic power in breast cancer. *Comput. Struct. Biotechnol. J*., 19, 4101–4109. 34527184 10.1016/j.csbj.2021.07.014 PMC8339292

[16] Cerami E. , Gao J., Dogrusoz U. et al. (2012) The cBio cancer genomics portal: an open platform for exploring multidimensional cancer genomics data. *Cancer Discov*., 2, 401–404. 22588877 10.1158/2159-8290.CD-12-0095 PMC3956037

[17] Goswami C.P. and Nakshatri H. (2014) PROGgeneV2: enhancements on the existing database. *BMC Cancer*, 14, 970. 10.1186/1471-2407-14-970 PMC4300843 25518851

[18] Chen X. , Miao Z., Divate M. et al. (2018) KM-express: an integrated online patient survival and gene expression analysis tool for the identification and functional characterization of prognostic markers in breast and prostate cancers. *Database*, 2018, bay069. 10.1093/database/bay069 PMC6041744 29992322

[19] Tang Z. , Kang B., Li C. et al. (2019) GEPIA2: an enhanced web server for large-scale expression profiling and interactive analysis. *Nucleic Acids Res*., 47, W556–W560. 31114875 10.1093/nar/gkz430 PMC6602440

